# CSF biomarkers of neuroinflammation and cerebrovascular dysfunction in early Alzheimer disease

**DOI:** 10.1212/WNL.0000000000006082

**Published:** 2018-08-28

**Authors:** Shorena Janelidze, Niklas Mattsson, Erik Stomrud, Olof Lindberg, Sebastian Palmqvist, Henrik Zetterberg, Kaj Blennow, Oskar Hansson

**Affiliations:** From the Clinical Memory Research Unit (S.J., N.M., E.S., O.L., S.P., O.H.), Department of Clinical Sciences, Lund University; Department of Neurology (N.M., S.P.) and Memory Clinic (E.S., O.H.), Skåne University Hospital; Institute of Neuroscience and Physiology (H.Z., K.B.), Department of Psychiatry and Neurochemistry, the Sahlgrenska Academy at the University of Gothenburg; Clinical Neurochemistry Laboratory (H.Z., K.B.), Sahlgrenska University Hospital, Sweden; Department of Molecular Neuroscience (H.Z.), UCL Institute of Neurology; and UK Dementia Research Institute at UCL (H.Z.), London.

## Abstract

**Objective:**

To measure CSF levels of biomarkers reflecting microglia and astrocytes activation, neuroinflammation, and cerebrovascular changes and study their associations with the core biomarkers of Alzheimer disease (AD) pathology (β-amyloid [Aβ] and tau), structural imaging correlates, and clinical disease progression over time.

**Methods:**

The study included cognitively unimpaired elderly (n = 508), patients with mild cognitive impairment (MCI, n = 256), and patients with AD dementia (n = 57) from the longitudinal Swedish BioFINDER cohort. CSF samples were analyzed for YKL-40, interleukin (IL)–6, IL-7, IL-8, IL-15, IP-10, monocyte chemoattractant protein 1, intercellular adhesion molecule 1 (ICAM-1), vascular adhesion molecule 1 (VCAM-1), placental growth factor, and fms-related tyrosine kinase 1 (Flt-1). MRI data were available from 677 study participants. Longitudinal clinical assessments were conducted in control individuals and patients with MCI (mean follow-up 3 years, range 1–6 years).

**Results:**

CSF levels of YKL-40, ICAM-1, VCAM-1, IL-15, and Flt-1 were increased during the preclinical, prodromal, and dementia stages of AD. High levels of these biomarkers were associated with increased CSF levels of total tau, with the associations, especially for YKL-40, being stronger in Aβ-positive individuals. The results were similar for associations between phosphorylated tau and YKL-40, ICAM-1, and VCAM-1. High levels of the biomarkers were also associated with cortical thinning (primarily in the precuneus and superior parietal regions) and with subsequent cognitive deterioration in patients without dementia as measured with Mini-Mental State Examination (YKL-40) and Clinical Dementia Rating Sum of Boxes (YKL-40, ICAM-1, VCAM-1 and IL-15). Finally, higher levels of CSF YKL-40, ICAM-1, and Flt-1 increased risk of development of AD dementia in patients without dementia.

**Conclusions:**

Neuroinflammation and cerebrovascular dysfunction are early events occurring already at presymptomatic stages of AD and contribute to disease progression.

β-Amyloid (Aβ) and tau pathology in Alzheimer disease (AD) is associated with a number of cellular reactions in the surrounding tissue. AD is accompanied by activation of microglia and astrocytes^1,2^ that affects the clearance and production of Aβ42,^3,4^ development and propagation of tau pathology,^5^ exacerbates neurodegeneration, and influences disease progression and severity.^6,7^ Genome-wide association studies have identified several single nucleotide polymorphisms in immune-related genes that are linked to increased risk of AD.^8^ AD is also associated with cerebrovascular changes that have been implicated in neuronal dysfunction and neurodegeneration.^9^ AD frequently co-occurs with cerebrovascular disease^10^ and these conditions have common risk factors including *APOE* ε4*,* hyperlipidemia, obesity, and others.^11^ At the same, studies have indicated that Aβ pathology may lead to secondary vascular damage including white matter lesions, microinfarcts, and microbleeds.^12^

Despite several lines of evidence supporting a role of neuroinflammation and cerebrovascular dysfunction in AD, further research is needed to elucidate how these changes are linked to Aβ and tau pathology and whether they represent an early phenomenon driving neurodegeneration and clinical progression, or merely downstream bystander effects of the disease. In the present study, we measured a panel of 11 inflammatory and vascular biomarkers in CSF of 508 cognitively unimpaired elderly and 313 patients with mild cognitive impairment (MCI) and AD dementia. We examined whether these biomarkers were associated with Aβ and tau pathology, cortical atrophy, rate of longitudinal cognitive decline, and risk of AD.

## Methods

### Standard protocol approvals, registrations, and patient consents

The study was approved by the Regional Ethics Committee in Lund, Sweden, and the patients or their relatives gave written informed consent.

### Study participants

The study population included 315 cognitively normal elderly participants (recruited from the population-based Malmö Diet Cancer Study^13^), 449 patients with mild cognitive complaints, and 57 patients with AD dementia enrolled consecutively at 3 memory outpatient clinics in Sweden between 2010 and 2014 (the prospective and longitudinal Swedish BioFINDER study; biofinder.se). Inclusion criteria for cognitively normal elderly were (1) age ≥60 years, (2) Mini-Mental State Examination (MMSE) 28–30 points at the screening visit, (3) absence of cognitive symptoms as evaluated by a physician, (4) fluency in Swedish, and (5) not fulfilling the criteria of MCI or any dementia. Individuals with (1) significant neurologic or psychiatric disease (e.g., stroke, Parkinson disease, multiple sclerosis, major depression), (2) significant systemic illness making it difficult to participate, or (3) significant alcohol abuse or (4) who were refusing lumbar puncture were excluded. Cognitively normal elderly underwent clinical assessments at baseline and 2-, 4-, and 6-year follow-up visits. The patients with mild cognitive complaints were thoroughly examined by physicians specialized in dementia disorders. The inclusion criteria were (1) cognitive symptoms, (2) not fulfilling the criteria for dementia, (3) MMSE 24–30 points, (4) age 60–80 years, and (5) fluent in Swedish. The exclusion criteria were (1) cognitive impairment that without doubt could be explained by another condition (other than prodromal dementia), (2) severe somatic disease, and (3) refusing lumbar puncture or neuropsychological investigation. These criteria resulted in a clinically relevant population where 193 individuals were classified as subjective cognitive decline (SCD) and 256 as MCI. The classification was based on a neuropsychological battery assessing the cognitive domains of verbal ability, visuospatial construction, episodic memory, and executive functions and the clinical assessment of a senior neuropsychologist. In agreement with US National Institute on Aging–Alzheimer's Association guidelines, cognitively normal individuals and study participants with SCD were included in the cognitively unimpaired control group.^14^ Patients with cognitive complaints underwent annual clinical assessments during 6-year follow-up. Patients with AD were required to meet the criteria for probable AD defined by National Institute of Neurological and Communicative Disorders and Stroke–Alzheimer’s Disease and Related Disorders Association.^15^ They were included when receiving their AD diagnosis and thereafter underwent annual clinical assessments. Cognitive progression was measured using the MMSE and Clinical Dementia Rating (CDR) based on thorough and standardized patient and informant rating scales, interviews, and cognitive tests. Study participants were categorized into groups with normal (Aβ−) and pathologic (Aβ+) CSF signature using the CSF Aβ42/Aβ40 ratio cutoff ≤0.1.^16^ Demographics are shown in table 1.

**Table 1:**
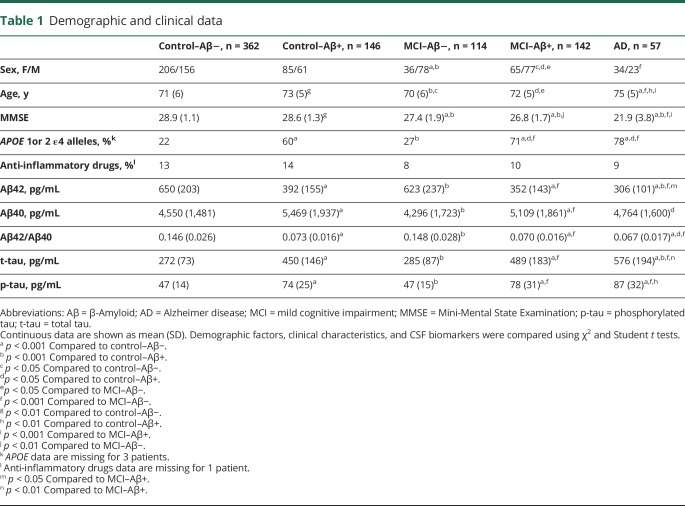
Demographic and clinical data

### CSF sampling and analysis

Collection of lumbar CSF samples was performed at the 3 centers with participants nonfasting and the samples were analyzed according to a standardized protocol.^17^ Following collection, the samples were centrifuged (2,000 *g*, +4°C, 10 minutes), 1 mL was aliquoted into polypropylene tubes (Sarstedt AG & Co., Nümbrecht, Germany), and aliquots were stored at −80°C. All CSF samples went through one freeze–thaw cycle before the analysis when 200 µL were further aliquoted into Lobind tubes (Eppendorf Nordic A/S, Hørsholm, Denmark).

CSF concentrations of the neuroinflammatory and cerebrovascular biomarkers (interleukin [IL]–6, IL-7, IL-8, IL-15, interferon-γ–induced protein 10 [IP-10], monocyte chemoattractant protein 1 [MCP-1], intercellular adhesion molecule 1 [ICAM-1], vascular adhesion molecule 1 [VCAM-1], placental growth factor [PlGF], and fms-related tyrosine kinase 1 [Flt-1]) were analyzed using ultrasensitive Mesoscale Discovery immunoassay and a customized V-PLEX kit. These biomarker assays were selected from the preconfigured V-PLEX Neuroinflammation Panel 1 Human Kit (combining proinflammatory, cytokine, chemokine, and angiogenesis panels) if the intra-assay and interassay coefficients of variation were below 20% and if the assays were sensitive enough for CSF analysis in our test samples/runs. CSF samples were analyzed with the customized kit according to the manufacturer's recommendations with one modification: for chemokine and proinflammatory panels, samples and calibrators were incubated overnight at +4°C.

CSF concentration of YKL-40 (chitinase-3-like protein 1), Aβ42, Aβ40, total tau (t-tau), and phosphorylated tau (p-tau) were measured using ELISA kits according to the manufacturer's recommendations (YKL-40, R&D Systems, Inc., Minneapolis, MN; Aβ42, Aβ40, t-tau, EUROIMMUN AG, Lübeck, Germany; p-tau, Fujirebio, Ghent, Belgium).

All analyses were performed using one batch of reagents and samples were randomized according to diagnosis across plates/runs to minimize the effects of run-to-run variation.

### MRI

A total of 735 controls and patients with MCI underwent MRI performed on a 3T Siemens (Erlangen, Germany) Trio system equipped with a standard 12-channel head coil. A single 3T MRI scanner (Siemens Medical Solutions) was used for all patients. Cortical thickness analysis modeling and volumetric estimations of cortical and subcortical brain regions were performed on structural T1 images with the FreeSurfer image analysis package version 5.3 (surfer.nmr.mgh.harvard.edu/). This software package was used for imaging intensity normalization, removal of nonbrain tissues, segmentation of cortical and subcortical brain regions into white and gray matter, spherical surface-based intersubject registration, which is based on the cortical surface curvature (sulci and gyri), and, finally, an automated parcellation of the cortical surface. FreeSurfer output was visually inspected for quality control. Of the original 735 participants, 717 had MRI segmentation from FreeSurfer. Quality control revealed 40 participants with segmentation errors and thus 677 were included in the final analysis.

### Statistical analysis

SPSS version 22 (IBM, Armonk, NY) and R version 3.3.1^18^ were used for statistical analysis. Two cases showed extremely high levels of multiple analytes and increased protein levels in CSF and were excluded from the study. IL-6, IL-8, and IP-10 were skewed (skewness 16.1, 9.1, and 14.1, respectively) and therefore ln transformed values were used in statistical analysis. Associations between CSF biomarkers of neuroinflammation and cerebrovascular changes and baseline characteristics (e.g., age, sex) were examined with the Pearson correlation and Student *t* tests. Some study participants were taking anti-inflammatory medications and there were also differences in age, sex, and *APOE* genotype between the diagnostic groups (table 1). Therefore, group differences in the biomarker levels were first tested with one-way analysis of variance and, when statistically significant, were further investigated in univariate general linear models (GLM) adjusting for the potential confounders with age, sex, *APOE* genotype, and anti-inflammatory medications included as covariates. For group comparisons, *p* values were corrected using the Bonferroni method. Associations between CSF biomarkers and tau were assessed using linear regression models. To determine whether the baseline biomarker levels were independent predictors of AD dementia, we used Cox proportional hazard regression models. All study participants were censored at their last follow-up visit or diagnosis of dementia. Because CSF biomarker scales differed considerably, standardized variables (continuous or tertiles) were used in Cox proportional hazard regression models. Associations between biomarkers and longitudinal MMSE and CDR Sum of Boxes (CDR-SB) were tested using linear mixed-effects models (with R v 3.2.3 and the lme4 package). These models had random intercepts and slopes for time and an unstructured covariance matrix for the random effects, and included the interaction between time and the biomarker as predictor. Associations between biomarkers and volumetric estimates of subcortical grey matter structures (corrected for total intracranial volume) and regional cortical thickness were studied using linear regression models adjusting for multiple comparisons with the false discovery rate procedure at a q value of 0.05. All regression models were adjusted for age, sex, and *APOE* genotype. The Query Design Estimate Contrast tool was used for GLM analysis at each vertex of the cortical surface. The model included sex and *APOE* ε4 carriers vs noncarriers as discrete factors and age as nuisance variable and cortical thickness as dependent variable. The results of the GLM analysis were corrected for multiple comparisons at the cluster level using the Monte Carlo simulation method for *p* cluster at *p* < 0.01 (*z* vertex 2.0).

### Data availability

Anonymized data will be shared by request from any qualified investigator for the sole purpose of replicating procedures and results presented in the article and as long as data transfer is in agreement with EU legislation on the general data protection regulation.

## Results

### Associations with demographic data

In the whole cohort, all CSF neuroinflammatory and cerebrovascular analytes except IL-6 correlated with age (data available from Dryad) (table 1) (doi.org/10.5061/dryad.5f82b32). The levels of IL-6, MCP-1, ICAM-1, VCAM-1, and PlGF were higher in men whereas Flt-1 levels were higher in women. There were no differences in YKL-40, IL-7, IL-8, IL-15, and IP-10 between men and women or in any of the biomarkers between *APOE* ε4 carriers and noncarriers (data available from Dryad) (table 1) (doi.org/10.5061/dryad.5f82b32). Study participants taking anti-inflammatory medications had higher concentrations of IP-10 but did not differ in the levels of the other biomarkers from those not taking anti-inflammatory medications (data available from Dryad) (table 1) (doi.org/10.5061/dryad.5f82b32).

### CSF biomarkers and amyloid pathology

To study possible associations between Aβ pathology and CSF biomarkers of neuroinflammation and cerebrovascular dysfunction, we first examined the effects of Aβ status on CSF biomarker levels in GLM, covaried for clinical diagnosis. We found that pathologic Aβ status was associated with higher levels of YKL-40, ICAM-1, VCAM-1, IL-15, and Flt-1 (all *p* < 0.001) in both control and MCI groups, and the interaction terms were negative (i.e., the effect of CSF Aβ status on the tested biomarkers was independent of clinical diagnosis). We did not observe any effect of Aβ status on the levels of IL-6, IL-7, IL-8, PlGF, IP-10, or MCP-1.

**Figure 1:**
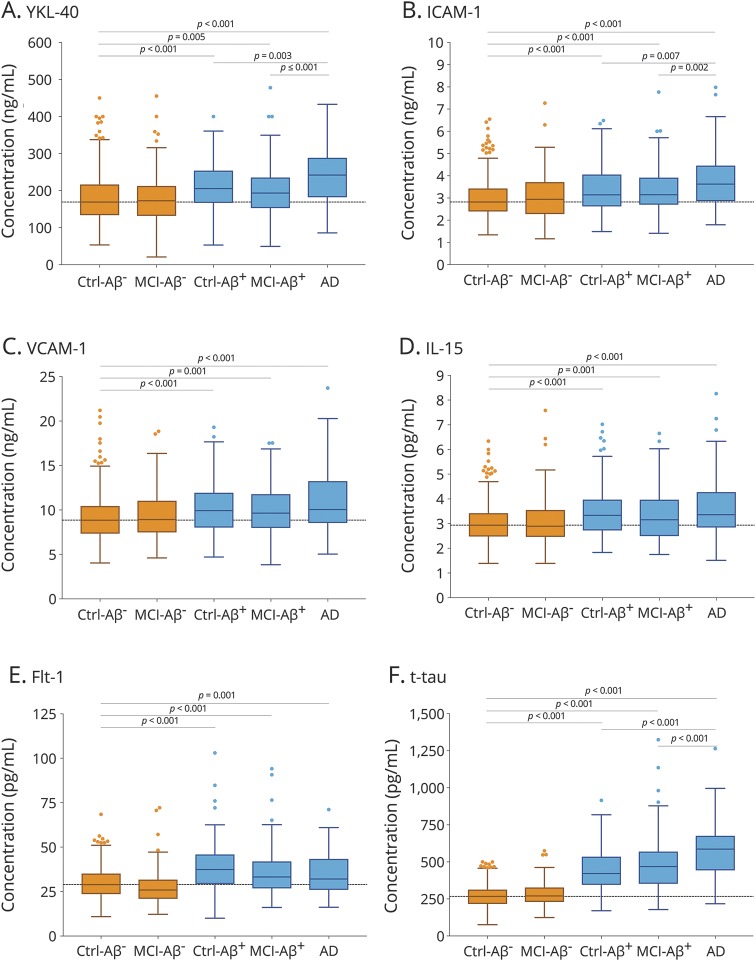
CSF biomarkers of neuroinflammation and cerebrovascular changes in diagnostic groups CSF levels of YKL-40 (A), intercellular adhesion molecule 1 (ICAM-1) (B), vascular adhesion molecule 1 (VCAM-1) (C), interleukin-15 (IL-15) (D), and fms-related tyrosine kinase 1 (Flt-1) (E) in cognitively unimpaired controls and patients with mild cognitive impairment (MCI) with normal (Ctrl–β-amyloid (Aβ)−, MCI–Aβ−, Aβ42/Aβ40 >0.1) and pathologic (Ctrl–Aβ+, MCI–Aβ+, Aβ42/Aβ40 ≤0.1) CSF Aβ status and patients with Alzheimer disease (AD) dementia. For comparison, CSF levels of total tau in the same diagnostic groups are shown in (F). The dotted lines indicate median levels in the Ctrl-Aβ− group. *p* Values are from one-way analysis of variance; statistical significance was set to *p* < 0.0071 (0.05/7) to account for Bonferroni correction. The significant findings were very similar when adjusting for the covariates (age, sex, *APOE* ε4 genotype, and anti-inflammatory medications), with the exception of ICAM-1, for which there were no differences between the groups with pathologic CSF Aβ status (Ctrl–Aβ+, MCI–Aβ+, and AD dementia).

To establish how early in the disease course neuroinflammation and cerebrovascular changes might occur, we compared control individuals with normal Aβ status (control–/Aβ−) to study participants with preclinical disease (controls with pathologic Aβ, control–/Aβ+), prodromal disease (MCI with pathologic Aβ status, MCI–Aβ+), and AD dementia. We also wanted to establish if the CSF levels of the biomarkers were associated with disease severity. For this, we studied differences in the biomarkers between the control–Aβ+, MCI–Aβ+, and AD groups. The results are summarized in figure 1. Compared with the control–Aβ− group, the CSF levels of YKL-40, ICAM-1, VCAM-1, IL-15, and Flt-1 were increased in all groups with pathologic Aβ status (control–Aβ+, MCI–Aβ+, and AD dementia) but not in MCI–Aβ−. Furthermore, YKL-40 levels were higher in AD dementia than in control–Aβ+ and MCI–Aβ+. There were no differences in VCAM-1, Il-15, and Flt-1 levels between the groups with pathologic Aβ status (control–Aβ+, MCI–Aβ+, and AD dementia). ICAM-1 was higher in AD dementia than in control–Aβ+ and MCI–Aβ+, but the differences between the groups were confounded by the effects of age, sex, *APOE* ε4 genotype, and anti-inflammatory medications and were no longer significant when these covariates were included in the regression models. The rest of the significant findings were very similar when adjusting for the covariates.

### CSF biomarkers and tau

**Figure 2:**
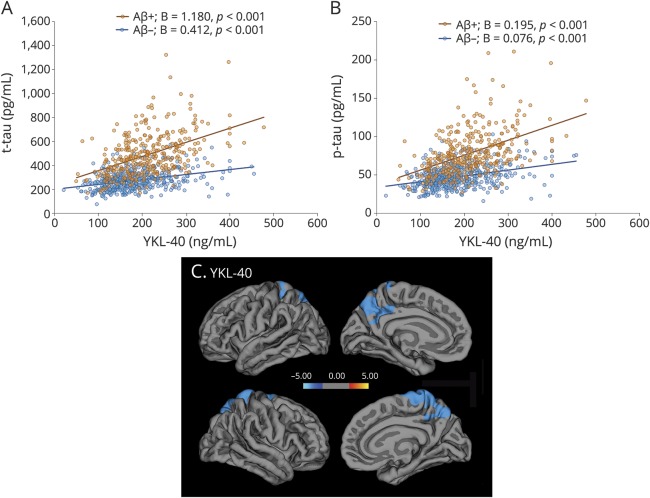
CSF biomarkers of neuroinflammation and cerebrovascular changes, CSF tau, and cortical atrophy (A, B) Association between CSF YKL-40 and total tau (t-tau) and CSF YKL-40 and phosphorylated tau (p-tau), respectively, in study participants with normal (β-amyloid [Aβ]−) and pathologic (Aβ+) CSF status. Unstandardized B coefficients and *p* values for slopes are from linear regression models. The results were very similar when adjusting for the covariates (age, sex, *APOE* ε4 genotype, and anti-inflammatory medications). (**C**) Associations between YKL-40 and cortical thickness. Voxel-wise regression analysis corrected for age, sex, and *APOE* ε4.

We next examined relationships between biomarkers of neuroinflammation and cerebrovascular dysfunction (YKL-40, ICAM-1, VCAM-1, IL-15, Flt-1) and CSF tau (t-tau and p-tau), and whether these relationships differed between individuals with normal and pathologic Aβ status. In the whole cohort, higher CSF levels of all biomarkers were associated with higher levels of t-tau and p-tau in both in Aβ-negative and Aβ-positive groups (table 2). YKL-40, ICAM-1, VCAM-1, IL-15, and Flt-1 interacted with Aβ status to predict t-tau (YKL-40 *p* < 0.001; ICAM-1 *p* = 0.040; VCAM-1 *p* = 0.002; IL-15 *p* = 0.043; Flt-1 *p* = 0.001). Increased levels of YKL-40, ICAM-1, VCAM-1, IL-15, and Flt-1 were associated with higher levels of t-tau in Aβ-positive than Aβ-negative people (figure 2A and table 2). We also found interaction effects between YKL-40, ICAM-1, VCAM-1, and Aβ status for p-tau (*p* < 0.001, *p* = 0.024, and *p* = 0.011) (figure 2B and table 2). Higher levels of IL-15 and Flt-1 were associated with higher p-tau levels, but these associations did not depend on Aβ status, i.e., were similar in Aβ-positive and Aβ-negative study participants. The differences in slopes between Aβ-positive and -negative individuals were particularly pronounced for YKL-40. The associations with t-tau and p-tau were similar in individual diagnostic groups (data available from Dryad) (tables 3 and 4) (doi.org/10.5061/dryad.5f82b32).

**Table 2:**
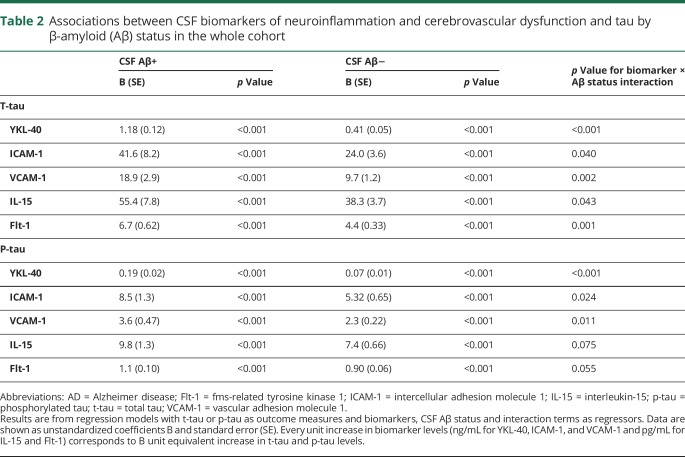
Associations between CSF biomarkers of neuroinflammation and cerebrovascular dysfunction and tau by β-amyloid (Aβ) status in the whole cohort

The significant associations between the biomarkers and tau were very similar when adjusting for the covariates (age, sex, *APOE* ε4 genotype, and anti-inflammatory medications) (data available from Dryad) (table 4) (doi.org/10.5061/dryad.5f82b32). Anti-inflammatory medications did not show significant effects in any of the statistical tests and only age, sex, and *APOE* ε4 were included as covariates in all subsequent statistical analysis.

### CSF biomarkers and gray matter atrophy

We performed voxel-wise regression analysis, which showed that high levels of CSF YKL-40 were associated with cortical thinning mostly in parietal areas including precuneus, posterior cingulate, and superior parietal cortices (figure 2C). Similar patterns were observed for the other biomarkers with further involvement of frontal regions (data available from Dryad) (figure 1) (doi.org/10.5061/dryad.5f82b32). In addition, higher levels of Flt-1 correlated with increased cortical thickness in insula, temporal, and cingulate cortices. In region of interest–based analyses, we found similar associations between CSF biomarkers and cortical thickness in some but not all areas that were significant in the voxel-wise regression models (data available from Dryad) (table 5) (doi.org/10.5061/dryad.5f82b32). The most consistent findings were associations between higher levels of the biomarkers and cortical thinning in the precuneus and superior parietal cortex (figure 2C) (data available from Dryad) (figure 1 and table 5) (doi.org/10.5061/dryad.5f82b32). When studying associations with subcortical gray matter volumes, we found that high levels of YKL-40 (but not other biomarkers) where associated with reduced volumes of left and right caudate and left amygdala (β = −0.092, *p* = 0.036; β = −0.124, *p* < 0.001; β = −0.102, *p* = 0.030; *false discovery rate*–adjusted *p* values).

### CSF biomarkers and AD progression

Associations between CSF biomarkers of neuroinflammation and cognitive decline were assessed in control participants and patients with MCI with repeated MMSE and CDR scores available at each visit (n = 757, average follow-up 3 years, range 1–6 years). Higher levels of YKL40 were associated with lower MMSE at baseline and with more rapid longitudinal decline in MMSE (table 3). Increased levels of YKL-40, VCAM-1, and IL-15 were associated with higher baseline CDR-SB. Increased levels of these biomarkers as well as ICAM-1 were also associated with more rapid longitudinal increase in CDR-SB (table 3).

**Table 3:**
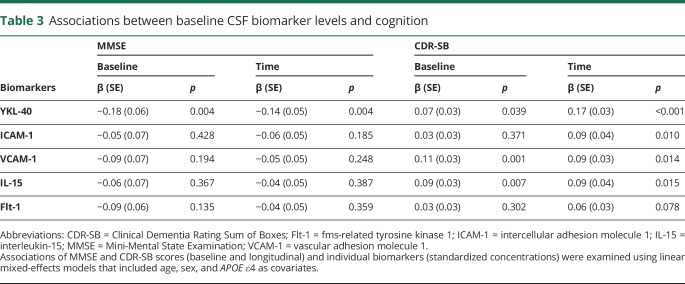
Associations between baseline CSF biomarker levels and cognition

**Figure 3:**
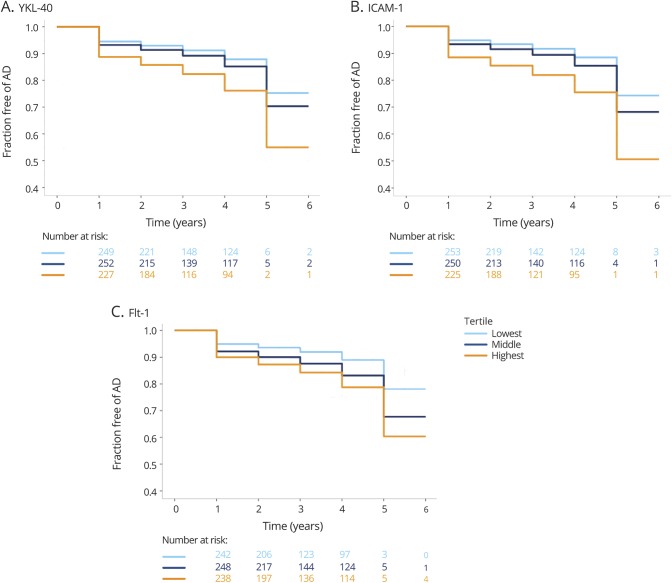
CSF biomarkers of neuroinflammation and cerebrovascular changes and progression to Alzheimer disease (AD) Kaplan-Meier curves of progression from cognitively normal and mild cognitive impairment (MCI) to AD for tertiles of YKL-40 (A), intercellular adhesion molecule 1 (ICAM-1) (B), and fms-related tyrosine kinase 1 (Flt-1) (C).

Follow-up diagnosis was available from 728 study participants who were cognitively unimpaired or had MCI at baseline, of whom 115 (15.8%) progressed to AD dementia (average follow-up 3 years, range 1–6 years). Cox regression analysis including age, sex, and *APOE* ε4 as covariates showed that YKL-40, ICAM-1, and Flt-1, but not VCAM-1 and IL-15, were independent predictors of time to AD diagnosis (table 4). Compared to the lowest tertiles, the highest tertiles of YKl-40 (*p* = 0.005), ICAM-1 (*p* = 0.001) and Flt-1 (*p* = 0.007), but not the middle tertiles, were associated with increased risk of AD dementia (figure 3).

**Table 4:**
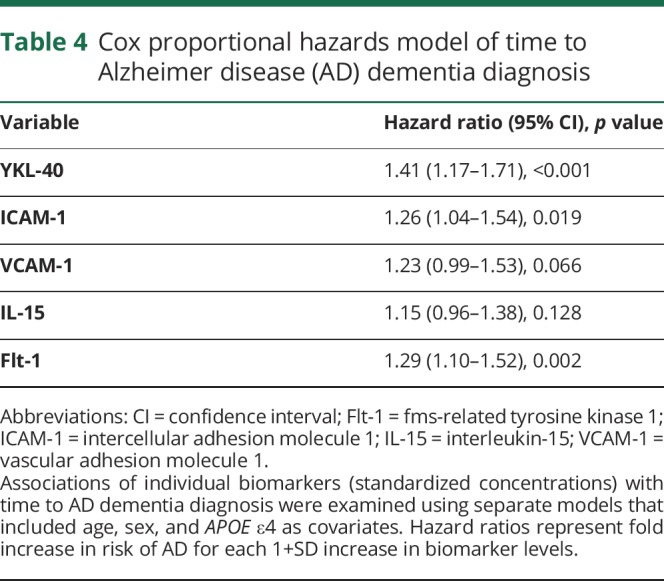
Cox proportional hazards model of time to Alzheimer disease (AD) dementia diagnosis

## Discussion

We found that CSF levels of 5 biomarkers of neuroinflammation and cerebrovascular dysfunction (YKl-40, ICAM-1, VCAM-1, IL-15, and Flt-1) were increased in AD already during the preclinical and prodromal stages. These biomarkers were associated with CSF tau, especially in Aβ-positive individuals. Further, increased concentrations of the biomarkers correlated with cortical thinning in the precuneus and superior parietal cortex. Finally, the longitudinal data suggested that higher levels of the neuroinflammatory and cerebrovascular biomarkers were associated with cognitive decline and increased risk of subsequent development of AD.

YKL-40 is a secreted glycoprotein that in the CNS is expressed by microglia and astrocytes^19,20^ and is considered one of the most promising biomarkers of neuroinflammation in AD. Our findings on YKL-40 agree with previous data showing increased CSF levels of this protein in preclinical, prodromal, and dementia stages of AD.^21–24^ A recent meta-analysis confirmed elevated levels of YKL-40 in AD dementia, although the association with AD was moderate compared with the core CSF AD biomarkers Aβ42, t-tau, and p-tau.^25^ Earlier studies have indicated that CSF YKL-40 is associated with tau pathology, since it may identify individuals with abnormal tau levels in CSF.^21,26^

We also showed that other CSF biomarkers of neuroinflammation and cerebrovascular dysfunction, including ICAM-1, VCAM-1, IL-15, and Flt-1, were associated with Aβ pathology, e.g., their levels were elevated in individuals with pathologic CSF Aβ status. ICAM-1 and VCAM-1 are adhesion molecules that are upregulated in endothelial cells under inflammatory conditions.^27^ In postmortem tissue from patients with AD, increased expression of ICAM-1 has been detected in plaques and astrocytes around plaques.^28^ High levels of ICAM-1 in temporal and cingulate cortices have been shown to correlate with larger Aβ plaque and neurofibrillary tangle loads in Lewy body disorder.^29^ Il-15 is a pleiotropic cytokine produced by many different cell types that regulates development, survival, and function of peripheral lymphocytes.^30^ While IL-15 could be found in both microglia and astrocytes, high expression by astrocytes has been shown to exacerbate tissue damage in multiple sclerosis and following brain ischemia.^31,32^ Flt-1 is one of the main receptors of the vascular endothelial growth factor (VEGF) family widely expressed in the vascular system.^33^ Apart from its role in the regulation of angiogenesis, Flt-1 (together with other members of VEGF family) has been implicated in glial cell development and adult neurogenesis.^34^ In animal models of cerebral ischemia, expression of Flt-1 is increased in astrocytes, endothelial cells, and neurons.^35^ Interestingly, upregulation of Flt-1 has been observed in the entorhinal cortical sections from human AD brain and in human microglia cells following treatment with Aβ42.^36^ One study has previously reported no difference in serum Flt-1 levels between patients with AD and healthy controls.^37^ Earlier investigations that have measured CSF and blood levels of ICAM-1, VCAM-1, and IL-15 in AD have produced conflicting results.^38–42^ This inconsistency in results could be partly due to heterogeneity of sample populations, various confounding factors, and small sample sizes. In the present study, using a large cohort of well-characterized patients, we show increased levels of ICAM-1, VCAM-1, IL-15, and Flt-1in AD at the preclinical, prodromal, and dementia stage, indicating that neuroinflammatory pathways are activated early in the disease course. These findings are in keeping with a recent report demonstrating that serum and CSF neuroinflammation biomarker signatures that included ICAM-1, VCAM-1, IL-15, and Flt-1 could identify individuals who had AD CSF profile (abnormal p-tau/Aβ42 ratio) in a group of patients with MCI.^43^ Of note, there was a considerable overlap in the CSF levels of YKL-40, ICAM-1, VCAM-1, IL-15, and Flt-1 between different groups in our study, suggesting that AD might be accompanied by neuroinflammation and cerebrovascular dysfunction in a subpopulation of patients. Future investigations assessing longitudinal changes in the biomarker levels are warranted in order to further clarify the roles of neuroinflammation and cerebrovascular dysfunction in AD progression including the development of tau pathology.

Previous studies have shown that higher CSF YKL-40 was associated with higher CSF tau, supporting a link between neuroinflammation and neurodegeneration.^21^ Accordingly, YKL-40 could distinguish patients with tau pathology from healthy controls.^26^ Here we report correlations between tau and several biomarkers of neuroinflammation and cerebrovascular dysfunction (YKL-40, ICAM-1, VCAM-1, IL-15, and Flt-1). Notably, the correlations were stronger in individuals with abnormal levels of CSF Aβ, indicating that the associations among neuroinflammatory, cerebrovascular, and neurodegenerative processes may be aggravated in the presence of Aβ pathology. The difference in the slopes was more pronounced for YKL-40 than for the other biomarkers (tables 2). A possible explanation for this finding could be that YKL-40 continued to increase with disease progression and reached its highest levels in AD dementia, whereas there were no differences in the levels of the other biomarkers among Aβ-positive controls, patients with prodromal AD, and patients with AD dementia. Thus, these data support the hypothesis that YKL-40 is associated with AD-related tau pathology in individuals with abnormal Aβ deposition.

High CSF levels of neuroinflammatory biomarkers correlated with cortical thinning predominantly in the precuneus and superior parietal cortex, but not in temporal areas, as has been reported for YKL-40 in a relatively smaller study.^44^ Both the precuneus and superior parietal cortex are parts of AD cortical signature that includes a set of cortical regions with consistent pattern of atrophy across different disease stages.^45^ Interestingly, the precuneus is one the nodes of the default mode network, where early deposition of Aβ occurs.^46^ Of relevance, PET studies have indicated increased microglia activation in the precuneus and parietal cortex in AD.^47,48^

In patients without dementia, higher levels of YKL-40 were associated with more rapid changes in MMSE and CDR-SB. At the same time, higher CSF levels of YKL-40, ICAM-1, VCAM-1, and IL-15 were related to more rapid increase in CDR-SB. Further, higher levels of the biomarkers were associated with increased risk of future AD. Two earlier reports have found that YKL-40 predicted progression to AD.^22,49^ Here we corroborate these findings in a considerably larger cohort. Moreover, we show that not only YKL-40 but also other biomarkers of neuroinflammation and cerebrovascular dysfunction are associated with cognitive impairment, thus suggesting that multiple neuroinflammatory and cerebrovascular factors might contribute to disease progression.

We demonstrate that AD is accompanied by longstanding changes in CSF levels of neuroinflammatory and cerebrovascular biomarkers, starting already at preclinical disease stages. These changes most likely reflect neuroinflammation and cerebrovascular dysfunction in the brain and our data indicate that these pathologic processes may contribute to tau pathology, neurodegeneration, cortical atrophy, and increased risk of AD dementia. Thus, our study provides evidence of the role of neuroinflammation and cerebrovascular dysfunction in AD and offers potential targets for novel therapeutic interventions.

## Glossary

Aβ: β-Amyloid
AD: Alzheimer disease
CDR: Clinical Dementia Rating
CDR-SB: Clinical Dementia Rating Sum of Boxes
Flt-1: fms-related tyrosine kinase 1
GLM: general linear model
ICAM-1: intercellular adhesion molecule 1
IL: interleukin
IP-10: interferon-γ–induced protein 10
MCI: mild cognitive impairment
MCP-1: monocyte chemoattractant protein 1
MMSE: Mini-Mental State Examination
p-tau: phosphorylated tau
PlGF: placental growth factor
SCD: subjective cognitive decline
t-tau: total tau
VCAM-1: vascular adhesion molecule 1
VEGF: vascular endothelial growth factor
